# The Dyspepsia of Phthisis

**Published:** 1895-03

**Authors:** 


*The Dyspepsia of Phthisis.* By W. SOLTAU FENWICK, M.D. Pp. xii., 203. London: H. K. LEWIS. 1894.

Dyspepsia, both as a cause and as one of the results of tubercular disease, deserves far more attention than it commonly receives. This book strikes out a new line, contains much interesting matter, and is likely to do good service to many a dyspeptic on the verge of phthisis, as well as to many of the victims of the secondary dyspepsia of tubercular disease.

It is well known that true tubercular ulceration of the gastric mucous membrane is exceedingly rare: in 2,000 autopsies on cases of phthisis at the Brompton Hospital, there were only two cases of true ulceration resulting from tubercle. The author's explanation of this rarity is, on the one hand the small amount of lymphoid tissue in the stomach, and on the other hand the hostility of acid secretion to bacillary growth; hence, even when introduced in large quantities into the stomach by means of the swallowed expectoration, the bacilli are rendered temporarily inert, and are passed on into the small bowel without having effected a permanent lodgment.

But it is more especially the gastro-enteritis of phthisis in its different varieties with which the author deals, and from the various facts cited he formulates the conclusion that "the gastritis is probably due to the chronic absorption of certain toxic substances which are manufactured in the pulmonary cavities." Several chapters are devoted to the relation of organic disease of the stomach to pulmonary tuberculosis, the dyspepsia preceding phthisis, the initial dyspepsia, and the terminal dyspepsia, including perforation.

One of the least defined of the varieties described is "the dyspepsia of strumous children." The author remarks that the tubercular diathesis is closely associated with various forms of neurosis, and it is therefore not unreasonable to suppose that the subjects of this disorder may occasionally exhibit an extreme irritability of the nervous mechanism of the digestive tract, which may show itself in the form of sudden and painful peristaltic waves. It must be easy, and often most convenient, to make a diagnosis of this malady, but equally difficult to establish the diagnosis on any accurate principle, seeing that pain in the abdomen is the one most constant and characteristic feature of the malady.

We are much astonished to find no mention of creasote, guaiacol, or their combinations, in the treatment of tubercular disorders of the alimentary canal.

